# Behind-the-Scenes Actors in Fertility: A Comprehensive Review of the Female Reproductive Tract Microbiome and Its Clinical Relevance

**DOI:** 10.3390/life15060916

**Published:** 2025-06-05

**Authors:** Anthi Papakonstantinou, Efthalia Moustakli, Anastasios Potiris, Athanasios Zikopoulos, Ermioni Tsarna, Chrysi Christodoulaki, Ioannis Tsakiridis, Themistoklis Dagklis, Periklis Panagopoulos, Peter Drakakis, Sofoklis Stavros

**Affiliations:** 1 Third Department of Obstetrics and Gynecology, University General Hospital “ATTIKON”, Medical School, National and Kapodistrian University of Athens, 12462 Athens, Greece; lina_iatros@yahoo.com (A.P.); apotiris@med.uoa.gr (A.P.); thanzik92@gmail.com (A.Z.); christodoulakichr@hotmail.com (C.C.); perpanag@med.uoa.gr (P.P.); pdrakakis@med.uoa.gr (P.D.); 2 Laboratory of Medical Genetics, Faculty of Medicine, School of Health Sciences, University of Ioannina, 45110 Ioannina, Greece; ef.moustakli@uoi.gr; 3 Second Department of Obstetrics and Gynecology, Aretaieion University Hospital, Medical School, National and Kapodistrian University of Athens, 11528 Athens, Greece; ermioni.tsarna@gmail.com; 4 Third Department of Obstetrics and Gynecology, General Hospital Ippokratio, Medical School, Aristotle University of Thessaloniki, 54642 Thessaloniki, Greece; iotsakir@gmail.com (I.T.); tdagklis@gmail.com (T.D.)

**Keywords:** microbiome, microbiota, female reproductive system, female infertility, *Lactobacillus*

## Abstract

The study of the microbiome has rapidly progressed over the past few decades, capturing the interest of both scientists and the general public. Nevertheless, there is still no widely agreed-upon definition for the term “microbiome” despite tremendous advances in our knowledge. The international scientific literature consistently underscores the difference between the human microbiome and human microbiota. Recent research has emphasized the importance of the female reproductive tract microbiome in fertility, impacting natural conception and assisted reproductive technologies (ARTs). This review explores the relationship between infertility and the microbiota of the female reproductive tract through a thorough evaluation of research papers and large-scale studies published up to 2024. The objective of this review is to critically assess current evidence on the role of the reproductive tract microbiome in female infertility and ART outcomes. Relevant papers were identified and analyzed through the electronic medical databases PubMed/MEDLINE and Scopus. A comprehensive synthesis of data from 36 original studies was performed, including observational, case–control, cohort, and randomized trials. By focusing on the vagina, cervix, and endometrium, this study offers a comprehensive overview of the microbiome throughout the female reproductive tract. RIF and poor reproductive outcomes are strongly linked to dysbiosis, which is characterized by a reduction in *Lactobacillus* species. *Lactobacillus crispatus*, in particular, plays a significant role in protecting against bacterial vaginosis and infertility. A thorough understanding of how the microbiome impacts fertility and the development of clinical strategies to improve reproductive outcomes requires standardized microbiome investigation techniques and larger, randomized trials that account for diverse patient characteristics.

## 1. Introduction

Humans have coevolved with various microbial organisms, including bacteria, viruses, fungi, and archaea [1,2]. These microbial communities are essential to preserving homeostasis since they inhabit almost every surface and cavity in the human body, including the female reproductive tract. Although the human genome has about 20,000 genes that code for proteins, the microbial genome is much more complicated, indicating that microbial genes mediate a sizable amount of the body’s immunological and physiological reactions [3].

**Figure 1:**
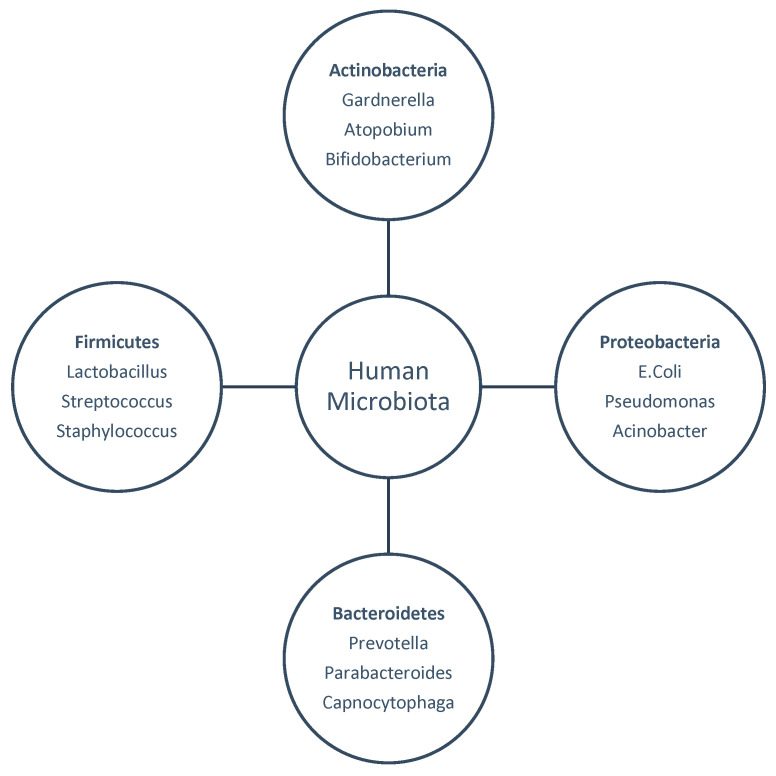
Overview of the female reproductive tract microbiota.

While the gut microbiome has long been a focus of human health research, increasing attention is being directed toward the microbial environments of the female reproductive tract. The Human Microbiome Project estimates that this area is home to about 9% of the human microbiome [4]. In the past, the uterus and upper female vaginal canal were considered to be lacking microbial populations. Recent research has refuted this notion, demonstrating that even in healthy people, bacterial translocation from the vagina to the cervix, uterus, fallopian tubes, and ovaries can happen. The microbial ecology of the reproductive tract and its connection to fertility have been reexamined in light of these discoveries [4]. Figure 1 presents the key bacterial phyla—Firmicutes, Actinobacteria, Proteobacteria, and Bacteroidetes—and representative genera found in the female reproductive tract.

The microbiota of the vagina, cervix, and uterus now appear to play critical roles in reproductive function, influencing processes such as endometrial receptivity, embryo implantation, and immune tolerance. Imbalances in the microbial ecosystem, or dysbiosis, have been implicated in preterm birth, preeclampsia, recurrent miscarriages, and infertility. However, despite these associations, the impact of microbial factors on clinical infertility has yet to be thoroughly acknowledged [4].

The inability to conceive after a year of unprotected sexual activity affects about 20% of women of reproductive age, according to the Centers for Disease Control and Prevention (CDC). One in four women worldwide has difficulty bringing their pregnancies to term. Infertility, recurrent pregnancy loss, or recurrent implantation failure are multifactorial entities, with the majority of research focusing on the immunologic and genetic causes [5,6,7]. However, the significance of the reproductive tract microbiome is one of the neglected etiological aspects that must be investigated as infertility becomes more common [8,9].

This review aims to compile and critically evaluate the existing evidence on the relationship between infertility and the microbiota of the female reproductive tract, particularly regarding the uterus, cervix, and vagina. Additionally, it investigates the potential impact of these microbial communities on outcomes associated with ART. The review follows international guidelines for the conduct and reporting of reviews, with the latest literature search completed in January 2024.

## 2. Materials and Methods

### 2.1. Literature Search Strategy

A comprehensive literature search was conducted to identify relevant studies on the relationship between the microbiome of the female reproductive tract and infertility. Scopus and PubMed/MEDLINE, two significant electronic databases, were thoroughly searched. The search method combined terms associated with infertility and the microbiome: (infertility, subfertility, sterility)* AND (microflora, microbiome, microbiota, microbiom, microbiot*). A total of 3596 articles were obtained, including 707 from Scopus and 2889 from PubMed/MEDLINE. Each database’s specific search approach is described in Table 1.

### 2.2. Research Questions

This thorough investigation aimed to address key issues concerning the role of the female reproductive tract microbiota in influencing assisted reproduction outcomes and fertility. The review specifically sought to determine whether the failure of assisted reproductive technologies (ARTs), recurrent implantation failure (RIF), or female infertility are linked to the composition of the vaginal, cervical, and endometrial microbiota. Women or couples experiencing infertility or unfavorable reproductive outcomes after ART procedures were among the target population. To emphasize microbiological differences, comparisons with fertile women were taken into consideration wherever possible. The studies that were included examined the reproductive tract microbiome as a potential exposure or intervention that could have an impact on infertility. Eligibility was limited to original, full-text research articles written in English. Abstracts, conference proceedings, opinion pieces, editorials, letters to the editor, review articles, and case reports were not included.

### 2.3. Sampling Methods

Meticulous sampling techniques are crucial for microbiome analyses of the female reproductive tract, as they prevent contamination and ensure anatomical accuracy. Vaginal samples are typically collected using sterile cotton swabs or cytobrushes to obtain vaginal secretions. Cervical biopsies, endocervical mucus, cervical swabs, or secretions collected using sterile swabs and cytobrushes are several approaches employed to obtain cervical microbiota samples. For uterine and endometrial microbiome analysis, samples are collected from endometrial biopsies or intrauterine fluid. These are usually obtained via embryo transfer catheters, sterile aspiration tubes, or double-lumen catheters, particularly during ART procedures. Early studies relied on basic techniques, such as the collection of vaginal or cervical fluids with cotton swabs, yet recent studies now utilize specialized swab kits and collection devices that minimize the risk of contamination from adjacent anatomical sites. Adherence to strict sampling protocols and proper storage of specimens after collection are essential for ensuring the reliability and validity of microbiome analyses.

### 2.4. Analytical Techniques

Analytical techniques used to describe the female reproductive tract’s microbiome have undergone substantial change. Histological analysis and microbiological cultures were two examples of traditional approaches that had limited sensitivity and scope. Modern research relies on a wide array of high-resolution molecular techniques to achieve comprehensive microbial profiling. Quantitative polymerase chain reaction (qPCR), next-generation sequencing (NGS), and 16S ribosomal RNA (rRNA) gene sequencing—specifically targeting the V3–V4 hypervariable regions—are extensively applied methodologies in microbial analysis. To distinguish between various microbial species, some researchers have also made use of cutting-edge technologies like IS-pro, a technique based on the 16S–23S rDNA region. Furthermore, metagenomics, transcriptomics, metabolomics, and whole genome sequencing (WGS) are being used more and more to offer in-depth understandings of microbial diversity, functional potential, and host–microbe interactions. These sophisticated analytical tools not only clarify the biological roles of microbial communities and their possible consequences for reproductive health and disease but also make it easier to characterize and taxonomically profile them.

## 3. Results

### 3.1. Study Selection

A total of 3596 records were found in the first literature search, including 2889 from PubMed/MEDLINE and 707 from Scopus. Following the initial screening and removal of duplicates, a total of 427 articles—93 from PubMed/MEDLINE and 334 from Scopus—were selected for further review. Eligibility was restricted to original research articles published in peer-reviewed journals in English.

Title screening resulted in the exclusion of 327 articles, leaving 100 for abstract review. Following the abstract screening phase, 36 articles were excluded based on the predefined eligibility criteria. A total of 64 full-text articles were assessed in detail, out of which 36 studies met all inclusion criteria and were included in the final qualitative synthesis.

**Figure 2:**
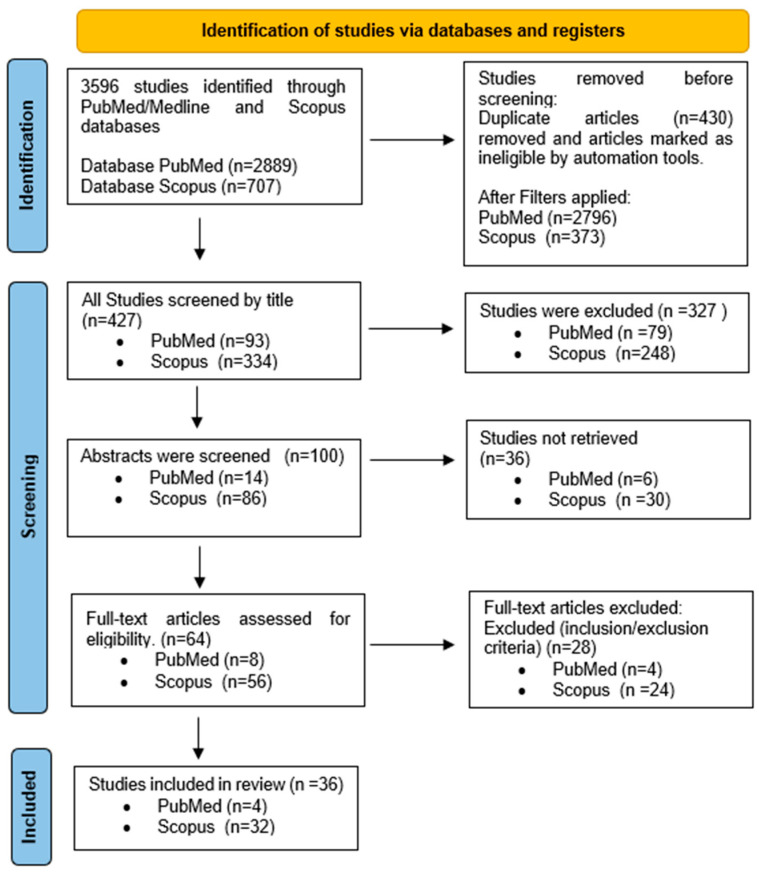
Flow diagram illustrating the selection process of the review.

The complete selection process is illustrated in the flow diagram of Figure 2, and the characteristics of the included studies are summarized in Table 2.

### 3.2. Microbiome of the Reproductive Tract in Infertility and ART Outcomes

The majority of the studies in this review focus on the vaginal microbiota of women undergoing IVF, who have recurrent implantation failure (RIF), are infertile, or have failed ART. Large sample sizes (>50 women) are common among these studies, and some are double-blind, randomized controlled trials, which yield more trustworthy results. The use of vaginal probiotics, especially *Lactobacillus*, in addressing dysbiotic microbiota before ART has also been the subject of a few investigations. Three research studies out of the 36 analyzed contained samples from infertile couples’ whole reproductive systems.

An interesting prospective study published in 2019 by Amato et al. in Italy included 23 infertile couples with idiopathic infertility. The study examined the vaginal and seminal microbiomes of the couples prior to ART methods. The results from NGS demonstrated a positive correlation between the vaginal microbiome (dominated by the species *L. crispatus*) and successful outcome of insemination in infertile women, with statistical significance (*p* = 0.002). Additionally, in the vaginal microbiome of infertile women, the most abundant microbial species were *Gardnerella vaginalis*, a pathogenic bacterium that contributes to the creation of vaginal dysbiosis, and Bifidobacterium breve, which is naturally present in the vaginal flora, both of which belong to the family *Bifidobacteriaceae*. Notably, in this study, the male participants’ sperm microbiome did not show differences in composition nor in insemination outcomes between infertile women and healthy fertile controls [13].

Another large cohort study by Vajpeyee et al., published in 2022 [38], included samples from the entire reproductive tract and comprised 197 infertile couples before their embryo transfer attempts. After analysis with NGS, disturbed microbiota were observed in the entire reproductive system of women who did not achieve a successful pregnancy after embryo transfer, with a characteristic decrease in *Lactobacillus* spp. and a simultaneous increase in pathogenic species such as *Prevotella*, *Gardnerella*, and *Atopobium*. The decrease in *Lactobacilli* coupled with an increase in pathogenic bacteria in the vaginal microbiome was strongly associated with IVF failure following embryo transfer, with statistical significance (*p* < 0.05) [38].

#### 3.2.1. Vaginal Microbiome and Its Role in Vaginitis/Dysbiosis

Vaginal dysbiosis, especially in the context of bacterial vaginosis (BV), has been linked to infertility in numerous studies. In a 2016 study, Haahr et al. [10] evaluated the vaginal microbiota of 130 infertile women undergoing IVF by comparing qPCR with Nugent Score criteria. According to the study, 28% of women were found to have an abnormal vaginal microbiome detected by qPCR, and a strong correlation was observed between this dysbiosis and reduced IVF success rates. A healthy microbiome was linked to *Lactobacillus* species, specifically *L. crispatus*, *L. jensenii*, and *L. gasseri*, whereas dysbiosis was linked to *L. iners*. The pregnancy rate was significantly lower in women with an abnormal vaginal microbiome (*p* < 0.05) [10].

In a cohort study by Campisciano et al. (2017) [11], a comparison of the vaginal microbiome of women with idiopathic and non-idiopathic infertility to healthy controls revealed a significant variation in microbial diversity. Although *L. crispatus* was less common in women with idiopathic infertility compared to healthy women, it is interesting to note that *L. iners* has sometimes been associated with a healthy microbiome [11]. Infertility, particularly idiopathic infertility, may still be affected by the dominance of *L. iners*, which is typically associated with dysbiosis.

A 2022 study by Ji et al. demonstrated that women with a normal vaginal microbiome had a higher embryo implantation rate and clinical pregnancy success after IVF compared to those with vaginal dysbiosis [34]. Furthermore, Haahr et al. (2019) found that an abnormal vaginal microbiome was associated with poor reproductive outcomes and lower chances of clinical pregnancy and live birth [15].

#### 3.2.2. Findings from Vaginal Microbiome Related to Recurrent Implantation Failure (RIF)

Vaginal dysbiosis has been closely associated with recurrent implantation failure (RIF). Ichiyama et al. (2021) reported that women with a history of recurrent implantation failure (RIF) exhibited significantly lower levels of *Lactobacillus* spp. in their vaginal and endometrial microbiomes, alongside higher quantities of pathogenic bacteria such as *Atopobium*, *Megasphaera*, *Gardnerella*, and *Prevotella* [24]. Diaz-Martinez et al. (2021) made similar observations, finding that recurrent implantation failure (RIF) was associated with poor ART outcomes due to elevated levels of *Streptococcus* and *Prevotella*, alongside a reduction in *Lactobacillus* levels [22].

Studies by Tanaka et al. (2022) and Bernabeu et al. (2019) further reinforced the notion that pathogens such as *Gardnerella vaginalis* and *Atopobium vaginae* are associated with poor reproductive outcomes, while a higher abundance of *Lactobacillus* species, particularly *L. crispatus*, is correlated with improved outcomes following embryo transfer [17,35]. Remarkably, women with RIF also had lower Shannon diversity indices, which were strongly associated with poor IVF outcomes, according to Kitaya et al. (2022) [16].

#### 3.2.3. Findings from Vaginal Microbiome Related to Antibiotic/Probiotic/Vaginal *Lactobacillus* Supplementation

Recent research has examined how probiotics and antibiotics may enhance the vaginal microbiome and reproductive outcomes. According to Eskew et al. (2021), prophylactic azithromycin had no significant effect on the vaginal microbiome or IVF success rates, suggesting that this strategy has limited effectiveness [27].

Conversely, research on the combination of probiotics and antibiotics has shown some potential. Haahr et al. (2020) conducted a randomized study to examine the effects of *L. crispatus* combined with clindamycin. The study is still in progress, and more findings are anticipated, even though only 9% of women with an aberrant microbiome became pregnant [21]. In a separate study, Lan et al. (2023) found that the combination of oral *Lactobacillus* capsules and metronidazole resulted in a more significant reduction in vaginal pH and improved reproductive outcomes [44].

The potential of probiotics in treating infertility may be restricted, as Jepsen et al. (2022) found that vaginal probiotics, including *L. gasseri* and *L. rhamnosus*, had little effect on modifying the vaginal microbiome or improving IVF outcomes [30].

#### 3.2.4. Findings from Vaginal Microbiome in Relation to IVF

The success of IVF is significantly influenced by the vaginal flora. According to a Kong et al. (2020) study, women who became pregnant following IVF had reduced levels of pathogens such as *Gardnerella vaginalis* and *Prevotella* spp. and increased abundances of *Lactobacillus* spp., especially *L. crispatus* [18]. The balance of *Lactobacillus* species plays a crucial role in successful reproduction, as demonstrated by a study from Azpiroz et al. (2021), which found that women with unsuccessful IVF outcomes had a higher *L. brevis*/*L. iners* ratio in their vaginal microbiome [23].

These findings emphasize the importance of maintaining a healthy vaginal microbiota, particularly with elevated *Lactobacillus* levels, to improve IVF success rates. To better understand the precise mechanisms by which the vaginal microbiota affects reproductive outcomes, more research is necessary.

### 3.3. Cervical Microbiome and Its Implications for Fertility

Despite emerging research indicating a potential link between the cervical microbiota and female infertility, it remains one of the least studied components of the female lower genital tract. Hao et al. [25] conducted a case–control study in China in 2021, including 124 infertile women before their first IVF attempt. The study found that in 84% of cervical smears, *Lactobacillus* was the dominant species, indicating its predominant role in the entire genital tract. Among women who did not achieve pregnancy after IVF, there was a dominance of *Firmicutes*, *Actinobacteria*, and *Bacteroidetes*, bacterial families associated with dysbiosis. Lactobacillus was negatively correlated with pathogens such as *Gardnerella* and *Dialister* and was positively correlated with estradiol. A significant difference in the diversity of cervical microbiota was observed between women who achieved pregnancy and those who did not, with statistical significance (*p* < 0.05). Clinical pregnancy outcomes were correlated with the cervical microbiota composition on the day of embryo transfer (*p* = 0.03) [25].

In 2021, Wang et al. [26] conducted another trial with 150 infertile women. According to the study, *Lactobacillus* was the most dominant species in both the cervical and vaginal microbiota, with percentages of 24.08% and 56.8%, respectively. Although no significant relationship was found between specific *Lactobacillus* species (e.g., *L. iners* and *L. crispatus*) and pregnancy outcomes (*p* > 0.05), a statistically significant relationship was found between *Prevotella* and unsuccessful IVF pregnancies (*p* = 0.004). Furthermore, ROC curve analysis showed that *Prevotella* and *Bifidobacterium* were predictive of unsuccessful pregnancies, while *Streptococcus* (*p* = 0.047) and *Fusobacterium* (*p* = 0.04) were predictive of negative pregnancy outcomes [26].

Participants in a 2022 study by Villani et al., which had 88 women receiving ART, were split into two groups according to their ART outcomes, either positive or negative. The study found that *Firmicutes* were the most dominant species in both groups, with higher percentages observed in the group with negative outcomes (82.2%) compared to the group with favorable outcomes (73.5%). The group with a positive outcome exhibited statistically significant (*p* < 0.05) lower levels of *Lactobacillus iners* and higher levels of *Lactobacillus* spp., particularly *L. crispatus*. This confirmed the positive impact of *L. crispatus* on fertility. Furthermore, the study found that successful ART outcomes were associated with a cervical microbiota predominantly composed of *Bifidobacteria* (*p* < 0.05). On the other hand, increased percentages of pathogens such as *Atopobium vaginae* (*p* = 0.01), *L. iners* (*p* = 0.023), *Firmicutes* (*p* = 0.033), and *Anaerococcus* (*p* = 0.023) were linked to unfavorable ART outcomes, suggesting the cervical microbiome as a potential marker for predicting reproductive success [32].

### 3.4. Endometrial Microbiome and IVF Success: The Role of Lactobacillus Dominance

Several studies conducted in the last 15 years have indicated that implantation failure and pregnancy loss may be exacerbated by an endometrial microbiome that has less than 90% *Lactobacillus* spp. dominance. In a pilot study conducted in Japan in 2018, Kyono et al. found that one hundred two infertile women and seven healthy controls had an endometrial microbiome dominated by *Lactobacillus* spp. (>90%); however, the abundance of *Lactobacillus* spp. was significantly lower in infertile women who needed IVF (38%) than in healthy controls (85.7%) (*p* = 0.001). *Prevotella*, *Atopobium*, *Streptococcus*, and *Gardnerella* were more common in infertile women [12].

A 2020 study by Vladislanovna et al. [19] in Russia found that infertile women with repeated unsuccessful IVF attempts had significantly lower levels of *Lactobacillus iners* (16.7% vs. 37.01%) and *Lactobacillus crispatus* (0.84% vs. 9.12%) in their endometrial microbiomes compared to healthy controls (*p* < 0.05). Infertile women had a lower percentage of *Lactobacillus* species (34.4%) than healthy controls (63%), indicating a beneficial relationship between IVF success and *Lactobacillus* species presence [19].

In 2022, Bednarska et al. [40] conducted a study in Poland that investigated endometrial and endocervical samples from 142 infertile women. They discovered that 33% of the samples had *Enterobacteriaceae* (*E. coli*) strains, while 57% of the samples had *Lactobacillus* and other normal microbiota strains. The study highlighted the variability of bacterial strains in the endometrial microbiome and the complex relationship between different species and IVF outcomes [40].

In an exploratory 2023 study conducted by Bui et al. in the Netherlands, the endometrial microbiota of women who had successful pregnancies following IVF was compared. The study revealed that *Gardnerella* levels were elevated in women with secondary infertility (*p* = 0.03), whereas the abundance of *L. crispatus* was significantly higher in women with live births (*p* = 0.002), especially in those with primary infertility. According to the study, *L. crispatus* may have a beneficial effect on IVF success, and a dysbiotic endometrial microbiome may be linked to subsequent infertility [43].

A 2022 study by Moreno et al. [4], involving 345 infertile women, found that women who had live births exhibited an abundance of *Lactobacillus* spp. greater than 85%, while women with poor reproductive outcomes had higher levels of pathogens such as *Gardnerella* and *Klebsiella* (*p* < 0.05) and lower levels of *Lactobacillus*. These findings reaffirmed how crucial endometrial *Lactobacillus* dominance is to the success of IVF [4].

#### 3.4.1. Endometrial Microbiome in Relation to Chronic Endometritis

One important factor influencing infertility and the result of IVF is chronic endometritis (CE). A 2019 study by Liu et al. [14] found that women with chronic endometritis had significantly different *Lactobacillus* abundances compared to those without the condition. Only 1.89% of women with CE possessed *Lactobacillus*, while women without CE had 80.7% (*p* < 0.001). *Actinobacteria*, *Gardnerella*, and *Prevotella* were the most prevalent pathogenic bacteria in the microbiomes of CE patients, indicating that the absence of *Lactobacillus* and the predominance of pathogens may hinder implantation and pregnancy [14].

Compared to 58% of women without CE, only 32% of women with chronic endometritis were pregnant in 2021 (*p* < 0.01), according to Chen et al. [29] *Atopobium* and *Gardnerella* dominated the microbiome of women with CE; however, *Lactobacillus* was more prevalent in those with a healthy endometrium. According to these results, the likelihood of a successful pregnancy is reduced by chronic endometritis, which is typified by dysbiotic microbiota [29].

#### 3.4.2. Endometrial Microbiome in Relation to Recurrent Implantation Failure (RIF)

RIF has been linked with dysbiosis of the endometrial microbiome in multiple studies. According to a 2022 study by Chen et al. [39], which included 111 infertile women (75 with RIF and 36 controls), there were significant differences in the endometrial microbiomes between RIF patients and controls. Gram-negative pathogen *Sphingomonas* dominated the microbiome of the RIF group, whereas *Lactobacillus* spp. predominated in the control group. Poor embryo implantation and decreased angiogenesis were linked to *Sphingomonas* presence [39].

In contrast, a 2022 study by Keburiya et al. [33] involving 130 women with infertility and RIF found no statistically significant differences between the RIF and control groups in terms of *Lactobacillus* abundance or the presence of opportunistic pathogens. Dysbiosis may not be a reliable indicator of RIF outcomes, as the study found that although RIF patients had somewhat lower *Lactobacillus* levels (73%) than those who achieved pregnancy (90%), these differences were not statistically significant (*p* > 0.05) [33].

## 4. Discussion

This comprehensive study examined the role of the microbiome in female infertility, particularly in ART, by reviewing research published from 2016 to 2024. A total of 36 publications were included, all of which met the criteria for original research. Of these, observational studies accounted for 36%, case–control studies 33%, cohort studies 25%, and a few randomized controlled trials 5.5%. Although most of the research focused on women aged 25 to 40, only half of the studies provided a specified age range, and this information was reported inconsistently [45].

The demographic characteristics and the country of origin of the studies were generally included, but the country of origin of the women themselves was often omitted. Notably, the majority of the studies (50%) were conducted in Asia, while the remainder were in European countries. This highlights the need for more research in this area, particularly in South America and Africa, where varied communities are underrepresented. This would provide a clearer understanding of how various factors, including lifestyle, socioeconomic status, and geographic location, influence the microbiome and its association with female infertility [46].

Most studies had relatively small sample sizes, with the majority involving 20 to 120 women. Larger sample sizes, ranging from 200 to 300 participants, were used in just three studies. Vaginal fluid was the most commonly used sample for microbiome analyses (48%), and all studies concentrated on women with secondary infertility. Other sample types included cervical swabs (11.5%), endometrial biopsies (7.7%), and endometrial fluid (32.7%). The fact that no studies utilized ovarian, peritoneal, or fallopian tube fluid samples indicates that these areas are still not well understood [47,48].

Microbiome analysis methods were predominantly next-generation sequencing (NGS), which was used by 83% of studies due to its ability to provide comprehensive, reliable results in a relatively short period. Other methods used included quantitative PCR (14%) and culture-based techniques (3%). Next-generation sequencing (NGS) enables direct DNA sequencing from samples, providing reliable data that enhances the validity of microbiome studies [49].

The majority of these research findings are consistent with the notion that fertility outcomes are significantly influenced by the composition of the female reproductive tract microbiome, namely the prevalence of *Lactobacillus* species. Over 90% of the studies demonstrated that a dysbiotic microbiome—characterized by a reduced proportion of *Lactobacillus* and an overabundance of pathogenic bacteria—was linked to poor reproductive outcomes, including ART failure and infertility. Studies examining the vaginal microbiome also found a significant association between *Lactobacillus* dominance and improved implantation and pregnancy rates, while a reduction in *Lactobacillus* was linked to implantation failure and ART failure [45].

While most research focuses on bacterial communities, it is crucial to remember that fungi, particularly Candida albicans, also live in the female reproductive system. *C. albicans* is a commensal fungus that affects about 20–30% of healthy women without symptoms. Although generally harmless, changes in the vaginal environment, such as hormonal changes, antibiotic usage, or immunological suppression, can cause fungal overgrowth and contribute to vaginal dysbiosis. New research indicates that imbalances in fungal populations may indirectly affect fertility by disturbing microbial equilibrium and triggering inflammation. Future studies should explore the mycobiome more thoroughly to assess its potential role in assisted reproductive technology (ART) outcomes and overall female reproductive health [50].

**Figure 3:**
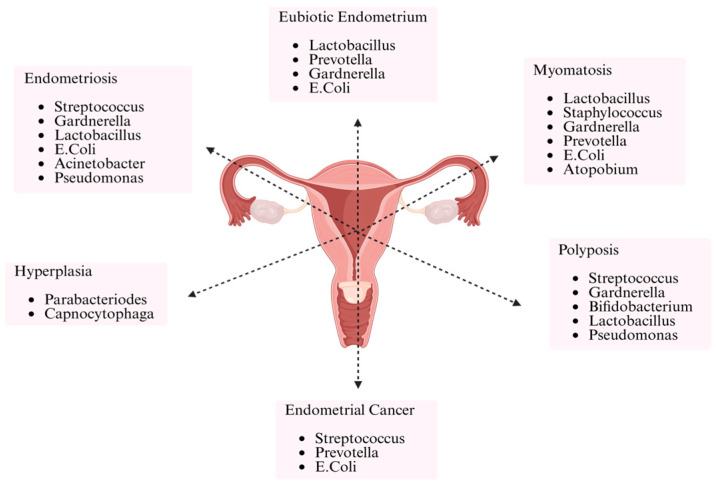
Microbial profiles associated with eubiotic and pathological endometrial conditions. The diagram illustrates key bacterial genera identified in the endometrium of women with normal (eubiotic) microbiota and those with endometriosis, hyperplasia, polyposis, myomatosis, and endometrial cancer, highlighting overlaps and condition-specific associations.

Recently, there has been growing evidence linking changes in the endometrial microbiome to several gynecological diseases, including endometriosis, hyperplasia, polyposis, myomatosis, and even endometrial cancer, in addition to fertility-related outcomes. Both eubiotic and pathological endometrial states are associated with distinct bacterial genera. Interestingly, genera such as *Gardnerella*, *E. coli*, and *Lactobacillus* are found in both healthy and pathological states, indicating that their impact may vary based on microbial context and relative abundance [51,52]. The clinical significance of microbial profiling in infertility and broader gynecological diagnostics is illustrated in Figure 3.

It is interesting to note that some research has indicated that gut microbiota may also have an impact on the uterine microbiota, which could lead to disorders like RIF. With pregnancy rates ranging from 6% to 9% after embryo transfer, the link between aberrant vaginal microbiota (AVM) and poor clinical pregnancy outcomes in IVF is becoming more apparent, even though research on this topic is still in its infancy. Ascending infections are believed to be the biological process underlying this association, which hinders effective embryo implantation [53].

The reported prevalence of AVM ranges from 4% to 38%, reflecting considerable variation in detection and diagnostic methods. This highlights the importance of establishing consistent diagnostic standards. The possibility of detecting and treating AVM presymptomatically remains unclear. Early intervention may lead to better ART outcomes in patients with dysbiosis, but more research is needed, as it could become a crucial approach to enhancing reproductive success.

In addition to microbial dysbiosis, hormonal imbalances and inflammatory responses in the female reproductive system can have a substantial impact on the microbiome. Estrogen and progesterone levels, for example, influence the vaginal and endometrial microbiota by affecting glycogen availability and epithelial barrier function. Similarly, persistent inflammation or immunological dysregulation in the genital tract can generate conditions that promote the growth of harmful bacteria while affecting the stability of beneficial microbiomes. Despite the discovery of these factors, their specific interactions with the microbiome in the context of infertility remain unknown and require further exploration [54].

More research is required to fully understand the endometrial, cervical, and vaginal microbiomes’ potential as infertility biomarkers, even if existing data indicate that they are important in female infertility and ART outcomes. Additionally, the impact of presymptomatic microbiome screening and treatment warrants exploration to improve reproductive outcomes. It is crucial to establish whether these microbes can be included as routine diagnostic tools for women undergoing ART.

## 5. Conclusions

This review emphasizes the significance of the microbiome in the female reproductive system and its potential role in influencing fertility, particularly concerning IVF success. RIF and poor reproductive outcomes are strongly linked to dysbiosis, which is characterized by a reduction in *Lactobacillus* species. *Lactobacillus crispatus*, in particular, plays a significant role in protecting against bacterial vaginosis and infertility. The diversity of *Lactobacillus* species and their precise role in fertility remain unclear. A thorough understanding of how the microbiome impacts fertility and the development of clinical strategies to improve reproductive outcomes require standardized microbiome investigation techniques and larger, randomized trials that account for diverse patient characteristics.

## 6. Strengths and Limitations

This review highlights consistent trends linking microbiome composition to fertility and ART outcomes, offering a comprehensive synthesis of data from 36 original studies, including observational, case–control, cohort, and randomized trials. Alongside its strict compliance with international review standards and carefully structured literature selection process, this review is notably strengthened by the inclusion of recent high-throughput sequencing studies, which significantly improve data reliability. By focusing on the vagina, cervix, and endometrium, this study offers a comprehensive overview of the microbiome throughout the female reproductive tract.

Comparability may be impacted by factors such as the geographic concentration of research in Asia and Europe, the generally small sample sizes, and the variation in sampling and analytical methods. Additionally, considerable variability exists in microbiota assessment methodologies across the included studies. Differences in sample collection techniques (for example, vaginal swabs vs. endometrial biopsies), DNA extraction protocols, sequencing platforms, and bioinformatic pipelines can all have a significant impact on microbial taxonomic detection and quantification. This methodological variation hinders direct comparisons between research and may lead to contradictory results. Standardized protocols for microbiome research in reproductive health are critical to improving repeatability and data integration across studies.

Furthermore, the paucity of long-term follow-up data and the underrepresentation of specific reproductive tract regions limit the findings’ generalizability. However, this study is one of the first thorough attempts to organize the available data on the effects of the female reproductive tract microbiome on fertility, emphasizing its potential as a therapeutic target in assisted reproductive technologies as well as a diagnostic biomarker.

## Figures and Tables

**Table 1:** Search strategy used in the selected electronic databases.

Database	Number of Studies Retrieved	Search Strategy
PubMed/MEDLINE	2889	(((microflora) OR (microbiome)) OR (microbiota)) OR (microbiom*)) OR (microbiot*)) AND (((infertility) OR (subfertility)) OR (sterility))
Scopus	707	TITLE-ABS-KEY ((microbiome) OR (microbiota) OR (microflora) OR (microbiom*) OR (microbiot*)) AND ((infertility) OR (subfertility) OR (sterility))

**Table 2:** Summary table of the articles included in the review.

Study/Authors	Country	Patient Population	Sample Type	Diagnostic Method
Haahr et al. (2016) [10]	Denmark	30 infertile women undergoing IVF	Vaginal fluid	qPCR, Nugent Score
Campisciano et al. (2017) [11]	Italy	96 idiopathic infertile women, 96 fertile controls	Vaginal and cervical fluid	NGS (16s rRNA V3)
Kyono et al. (2018) [12]	Japan	102 infertile women (79 IVF, 23 non-IVF), 7 fertile controls	Vaginal and endometrial fluid	NGS (16s rRNA V4)
Amato et al. (2019) [13]	Italy	23 infertile couples undergoing IUI	Vaginal and seminal fluid	NGS (16s rRNA V4)
Liu, Y., et al. (2019) [14]	China	130 infertile women (12 with chronic endometritis, 118 without)	Endometrial fluid and biopsy	PCD, PCR, NGS (16s rRNA V4)
Haahr et al. (2019) [15]	Denmark	75 infertile women before embryo transfer	Vaginal fluid	qPCR, NGS (16s rRNA V4)
Kitaya et al. (2019) [16]	Japan	28 infertile women with RIF, 18 women with first IVF	Vaginal and endometrial fluid	NGS (V3–V4 of 16s rRNA)
Bernabeu et al. (2019) [17]	Spain	31 infertile women undergoing ART	Vaginal fluid	NGS (V3–V4 of 16s rRNA)
Kong et al. (2020) [18]	China	475 infertile women (238 pregnant, 237 not pregnant after IVF)	Vaginal fluid	PCR (V4 of 16s rRNA)
Vladislavnova et al. (2020) [19]	Russia	22 women with >2 IVF failures, 20 healthy controls	Endometrial fluid	NGS (V3–V4 of 16s rRNA)
Zhao et al. (2020) [20]	China	22 women with >2 IVF failures, 20 healthy controls	Endometrial fluid	NGS (V3–V4 of 16s rRNA)
Haahr et al. (2020) [21]	Denmark	111 infertile women undergoing IVF	Cervical fluid	NGS qPCR
Diaz-Martinez et al. (2021) [22]	Spain	48 infertile women prior to ARTs	Vaginal and cervical fluid	NGS V3–V4 16sRNA
Azpiroz et al. (2021) [23]	Argentina	287 infertile women with multiple IVF attempts, 20 fertile controls	287 infertile women with multiple IVF attempts and 20 fertile controls	NGS miRNA PCR
Ichiyama et al. (2021) [24]	Japan	89 infertile women with RIF, 17 fertile women	Cervical and vaginal fluid	NGS 16sRNA
Hao et al. (2021) [25]	China	124 infertile women undergoing IVF	Vaginal and seminal fluid	NGS (16s rRNA V4)
Wang et al. (2021) [26]	China	150 infertile women prior to their first IVF-ET	Vaginal and cervical fluid	NGS (16s rRNA V4)
Eskew et al. (2021) [27]	USA	27 infertile women with RIF, 12 non-IVF controls	Cervical fluid	NGS (16s rRNA)
Karaer et al. (2021) [28]	Turkey	223 infertile women prior to ART	Vaginal and seminal fluid	NGS (16s rRNA V4)
Chen et al. (2021) [29]	China	94 infertile women (25 with chronic endometritis, 69 without)	Endometrial fluid	NGS (V4 of 16s rRNA)
Moreno et al. (2022) [4]	Spain	345 infertile women before embryo transfer	Endometrial fluid	NGS (16s rRNA V4)
Jepsen et al. (2022) [30]	Denmark	74 infertile women before ART	Vaginal fluid	NGS (16s rRNA V4)
Iniesta et al. (2022) [31]	Spain	17 infertile couples	Vaginal, seminal, endometrial fluid, and plasma	NGS (16s rRNA V4)
Villani et al. (2022) [32]	Italy	88 infertile women	Cervical smear	NGS (V3–V4 of 16s rRNA)
Keburiya et al. (2022) [33]	Russia	130 infertile women	Endometrial fluid and cervical smear	NGS (V3–V4 of 16s rRNA)
Ji et al. (2022) [34]	China	229 infertile women undergoing frozen embryo transfer	Vaginal smear	NGS (16s rRNA V4)
Tanaka et al. (2022) [35]	Japan	123 infertile women with and without chronic endometritis	Endometrial biopsy and vaginal fluid	NGS (16s rRNA V4)
Lull et al. (2022) [36]	Estonia	25 infertile women with first IVF failure	Endometrial fluid and biopsy	NGS (V3–V4 of 16s rRNA)
Patel et al. (2022) [37]	India	20 women with unexplained infertility, 11 fertile controls	Vaginal and fecal fluid	NGS (16s rRNA V2–V3)
Vajpeyee et al. (2022) [38]	India	197 infertile couples before IVF	Vaginal, follicular, endometrial, and seminal fluid	NGS (16s rRNA V4)
Chen et al. (2022) [39]	China	75 women with RIF and 36 healthy controls	Endometrial fluid	NGS (16s rRNA V4)
Bednarska-Czerwinksa et al. (2022) [40]	Poland	142 infertile women before IVF	Endometrial and cervical fluid	NGS (16s rRNA V4)
Sezer et al. (2022) [41]	Turkey	26 infertile women, 26 healthy controls	Vaginal and endometrial smear	Real-time PCR
Zou et al. (2023) [42]	China	141 infertile women with RIF	Vaginal fluid	NGS 16s rRNA
Bui et al. (2023) [43]	The Netherlands	141 infertile women with first IVF/ICSI cycle	Vaginal fluid	NGS V1–V2 16sRNA
Lan et al. (2023) [44]	China	100 infertile women undergoing IVF and 50 healthy controls	Cervical fluid	Calfrolferia Gram-negative anaerobic

## Data Availability

No new data were created or analyzed in this study.
